# A possible association between statin use and improved *Clostridioides difficile* infection mortality in veterans

**DOI:** 10.1371/journal.pone.0217423

**Published:** 2019-05-28

**Authors:** Jacqueline R. Argamany, Grace C. Lee, Bryson D. Duhon, Amina R. Zeidan, Eric H. Young, Kelly R. Reveles

**Affiliations:** 1 College of Pharmacy, The University of Texas at Austin, Austin, TX, United States of America; 2 Pharmacotherapy Education and Research Center, UT Health San Antonio, San Antonio, TX, United States of America; University of British Columbia, CANADA

## Abstract

*Clostridioides difficile* infection (CDI) is the most common cause of nosocomial diarrhea and places a significant burden on patients and the health care system. Statins could lead to improvements in CDI clinical response due their pleiotropic effects, including immunomodulatory and lipid-lowering effects; however, few studies have assessed this association. The primary objective of this study was to compare CDI health outcomes in statin users and non-users in a national cohort of patients. This was a retrospective cohort study of all adult CDI patients receiving care from the Veterans Health Administration from 2002 to 2014. Patients were divided into two groups based on statin exposure 90 days prior to and during their first CDI encounter. CDI health outcomes, including mortality and CDI recurrence, were compared using a propensity-score matched cohort of statin users and non-users and multivariable logistic regression. A total of 26,149 patients met study inclusion criteria, of which 173 statins-users and 173 non-users were propensity score matched. Thirty-day mortality was significantly lower among statins users with CDI (12.7%) compared to non-users (20.2%) (aOR 0.34; 95% CI 0.16–0.72). Sixty-day CDI recurrence was non-significantly lower among statin-users (9.0%) compared to non-users (16.6%) (aOR 0.68; 95% CI 0.29–1.59). In this nationally-representative study of veterans with CDI, statin use was associated with lower 30-day mortality compared to non-use. Statin use was not associated with 60-day CDI recurrence.

## Introduction

*Clostridioides difficile* is clinically significant for its association with antibiotic-associated diarrhea.[1, 2] Specifically, *Clostridioides difficile* infection (CDI) is the most common cause of nosocomial diarrhea and more recently, the most common causative organism in healthcare-associated infections (HAIs) in the United States (U.S.).[3] Current treatment recommendations from the Infectious Disease Society of America (IDSA) include therapy with oral antibiotics, which can further disrupt the balance of normal gut microbiota.[4, 5] It can take weeks to months for patients to recover from infection and restore normal flora.[6] In that time, patients are susceptible to recurrent CDI, either by reinfection of *C*. *difficile* through spore germination and vegetative cell exposure or by relapse of the initial infection.[7] Due to the severity of CDI, several adjunctive treatments have been utilized.

A unique approach to the adjunctive treatment of CDI involves the use of statins, which are traditionally used for their myriad of effects on lipids and inflammatory pathways.[8–10] Statins are reversible, competitive inhibitors of 3-hydroxy-3-methyl-glutaryl-coenzyme A (HMG-CoA) reductase, the rate-limiting enzyme in cholesterol biosynthesis. Though traditionally used to treat hyperlipidemia, other beneficial aspects of statins include its anti-inflammatory, immunomodulatory, antioxidant, and antithrombotic effects. These effects may reduce the response of intracellular signaling pathways,[11] revealing a critical interception of inflammatory responses in CDI patients.[12, 13] Furthermore, the increase in inflammatory response experienced due to hyperlipidemia can increase the risk of negative clinical outcomes such as a recurrence of CDI and death, which is often seen in patients who suffer from inflammatory-related diseases, such as inflammatory bowel disease.[14–17]

Despite these potential benefits, limited data currently exist regarding the outcomes of statin users who develop CDI compared with non-users; therefore, the primary objective of this study was to compare CDI health outcomes in statin users and non-users in a national cohort of patients from a single-payer health system.

## Methods

### Study population and data collection

This was a retrospective cohort study of all CDI patients receiving care at any inpatient or outpatient Veterans Health Administration (VHA) facilities in the U.S., and data for this study were obtained from the Veterans Affairs Informatics and Computing Infrastructure (VINCI). Eligible patients included adults age 18 to 89 years who had any inpatient or outpatient ICD-9-CM code for CDI (008.45) plus a positive stool test (toxin enzyme immunoassay or nucleic acid amplification test ± glutamate dehydrogenase) for CDI during the visit or within seven days of the visit from October 1, 2002 through September 30, 2014. We also included a one-year observation window preceding the visit (October 1, 2001 through October 1, 2002) to assess prior comorbidities and medication use. The cohort was limited to first episode CDI patients and excluded patients without active CDI treatment. The CDI encounter date, meaning the initial date of inpatient hospitalization or outpatient clinic visit during which CDI was diagnosed, was used as the index date for all variables unless otherwise noted. Data collection for patient demographics, comorbidities, and medication use have been described previously.[18] The institutional review boards at UT Health San Antonio and South Texas Veterans Health Care System Research and Development Committee approved this study under expedited review and waived the need for informed consent.

Patients were divided into two groups, statin users and non-users, based on statin exposure prior to and during the CDI encounter. Statin use was a composite variable, defined as patients meeting criteria for both prior and concomitant statin use. Prior statin users were defined as patients who filled at least one prescription for a statin medication in the 90 days prior to initial CDI encounter. Concomitant statin use represents documented statin use during a CDI episode. Non-users were defined as patients who had no prescription history of statins in the 90 days prior to initial CDI encounter or within 14 days of the CDI treatment start date. All statin products available in the U.S. were evaluated for prescription use (atorvastatin, fluvastatin, lovastatin, pitavastatin, pravastatin, rosuvastatin, and simvastatin).

To better differentiate the effect of statins beyond cholesterol control, we collected the patient’s most recent serum low-density lipoprotein (LDL) cholesterol level drawn in the year prior to first CDI encounter and controlled for the level in our analysis.

To minimize potential confounding from a healthy user effect, variables accounting for healthcare utilization were collected. These include: 1) ≥1 outpatient visit in the 90-days prior to initial CDI diagnosis, 2) hospitalization or surgery within the 90-days prior to initial CDI diagnosis, 3) receiving chronic dialysis therapy, and 4) residence in a long-term care facility (LTCF). These variables will account for a wider variety of patients at risk of exposure to *C*. *difficile* spores than is included in the surveillance definition for CO-HCFA CDI, and aim to reduce potential bias from the healthy user effect. History of aspirin and non-statin antilipemic agents (bile acid sequestrants, ezetimibe, fibric acids, and niacin) used in the 90 days prior to the initial CDI encounter was also collected.

Mortality was defined as death from any cause during inpatient hospitalization or within 30 days following CDI treatment discontinuation. Severe or complicated CDI was defined as the presence of at least one CDI severity indicator as described above. A recurrence was defined as a second outpatient or inpatient visit during which a patient received an ICD-9-CM code for CDI with a minimum three day gap between the visit and the end of active CDI therapy for the initial episode. As in previous studies, 60-day recurrence was used as a specific endpoint.[18]

### Data and statistical analyses

Data extraction and variable creation were conducted using SAS Version 9.4 (SAS Institute, Cary, NC, USA). Propensity score matching was performed using STATA 14 (StataCorp, College Station, TX, USA). All other data and statistical analyses were conducted using JMP 13 (SAS Institute, Cary, NC, USA).

For the primary analysis of the matched cohort, we created a propensity score-matched cohort to account for the variables associated with indications for statin use. Propensity scores were created using a multivariable logistic regression model. We then performed nearest neighbor matching (1:1) with a caliper of 0.2. A total of 26 variables were included in the derivation propensity scores, including those outlined in Table 1, as well as fiscal year. Once the matched cohort was derived, an additional 25 variables that could have impacted CDI outcomes were entered into a multivariable logistic regression model in order to determine the risk of individual CDI outcome (Table 1). Variables with less than 5% of the cohort were not entered into the propensity score model or CDI outcomes models in order to improve model stability. Finally, an aOR and 95% CI was calculated using logistic regression for each CDI outcome. For the primary analysis of the risk of 60-day recurrence, we excluded those who died within 60 days of the end of treatment discontinuation for the initial episode to capture only those patients at risk for 60-day recurrence. This exclusion occurred prior to propensity score matching. As a sensitivity analysis, we repeated the analysis without excluding those who died within 60 days.

**Table 1:** Baseline characteristics of matched cohort.

Characteristic	Statin users n = 173	Non-users n = 173	P-value	SMD
Age (years), median (IQR)*	66 (62–75)	67 (62–76)	0.5693	0.0865
Male sex, n (%)*	169 (97.7)	169 (97.7)	1.0000	0.0000
White, n (%)*	133 (76.9)	127 (73.4)	0.7530	0.1025
Hispanic, n (%)*	9 (5.2)	11 (6.4)	0.6447	-0.1174
Priority group, median (IQR)*	5 (2–5)	5 (1–5)	0.2857	0.1084
LDL (mg/dL), median (IQR)*	83 (65–104)	85 (62–109)	0.4530	-0.1009
Smoker, n (%)*	60 (34.7)	57 (32.9)	0.7332	0.0427
Principal CDI, n (%)+	57 (32.9)	53 (30.6)	0.6442	0.0588
CDI type, n (%)+			0.3041	
CA-CDI	25 (14.5)	29 (16.8)		-0.0969
CO-HCFA-CDI	40 (23.1)	50 (28.9)		-0.01661
HCFO-CDI	108 (62.4)	94 (54.3)		0.1841
Comorbidities, n (%)				
Hypertension*	154 (89.0)	155 (89.6)	0.8619	-0.0334
Dyslipidemia*	157 (90.8)	156 (90.2)	0.8548	0.0369
Obesity*	54 (31.2)	55 (31.8)	0.9079	-0.0148
Myocardial infarction*	29 (16.8)	27 (15.6)	0.7703	0.0470
Congestive heart failure*	57 (32.9)	53 (30.6)	0.6442	0.0588
Peripheral vascular disease*	47 (27.2)	42 (24.3)	0.5385	0.0835
Cerebrovascular disease*	48 (27.7)	47 (27.2)	0.9041	0.0160
Dementia	3 (1.7)	5 (2.9)	0.4720	-0.2882
COPD*	55 (31.8)	53 (30.6)	0.8165	0.0297
Rheumatologic disease	4 (2.3)	2 (1.2)	0.4057	0.3886
Peptic ulcer disease	2 (1.2)	5 (2.9)	0.2444	-0.5149
Liver disease	5 (2.9)	8 (4.6)	0.3943	-0.2691
Diabetes*	105 (60.7)	103 (59.5)	0.8262	0.0266
Hemi-/paraplegia	11 (6.4)	8 (4.6)	0.4781	0.1857
Renal disease*	63 (6.4)	62 (35.8)	0.9109	0.0138
Neoplastic disease*	50 (28.9)	39 (22.5)	0.1757	0.1842
HIV/AIDS	4 (2.3)	2 (1.2)	0.4057	0.3886
GERD*	55 (31.8)	53 (30.6)	0.8165	0.0297
Transplant	6 (3.5)	4 (2.3)	0.5196	0.2301
Inflammatory bowel disease	4 (2.3)	5 (2.9)	0.7353	-0.1263
Irritable bowel syndrome	1 (0.6)	3 (1.7)	0.3036	-0.6121
Concomitant infections, n (%)				
Bacteremia+	16 (9.3)	14 (8.1)	0.7023	0.0806
Pneumonia+	30 (17.3)	36 (20.8)	0.4114	-0.1242
Skin infection+	19 (11.0)	17 (9.8)	0.7247	0.0684
Intra-abdominal infection+	12 (6.9)	10 (5.8)	0.6593	0.1073
Device-related infection	5 (2.9)	3 (1.7)	0.4720	0.2882
Acute respiratory infection	7 (4.1)	4 (2.3)	0.3550	0.3184
Endocarditis	2 (1.2)	1 (0.6)	0.5583	0.3854
Urinary tract infection	6 (3.5)	3 (1.7)	0.3064	0.3920
CDI severity indicators, n (%)				
Any severity indicator	135 (78.0)	128 (74.0)	0.3779	0.1226
ICU admission	2 (1.2)	3 (1.7)	0.6513	-0.2268
Sepsis/septicemia+	39 (22.5)	29 (16.8)	0.1755	0.2030
Shock+	13 (7.5)	9 (5.2)	0.3769	0.2164
Acute renal failure+	78 (45.1)	66 (38.2)	0.1904	0.1577
Ileus	7 (4.1)	5 (2.9)	0.5559	0.1921
Perforated intestine	3 (1.7)	2 (1.2)	0.6513	0.2268
WBC ≥15,000 cells/μL+	81 (46.8)	69 (39.9)	0.1928	0.1560
CRP ≥160 mg/L	4 (2.3)	6 (3.5)	0.5196	-0.2301
Albumin <2.5 g/dL+	69 (39.9)	65 (37.6)	0.6589	0.0537
SCr >1.5 mg/dL+	43 (24.9)	56 (32.4)	0.1216	-0.2037
Prior medications, n (%)				
Antibiotics+	94 (54.3)	102 (59.0)	0.3854	-0.1039
GAS drugs+	115 (66.5)	98 (56.6)	0.0600	0.2299
Narcotics+	78 (45.1)	66 (38.2)	0.1904	0.1577
Anti-diarrheals+	8 (4.6)	13 (7.5)	0.2581	-0.2846
Bowel prep+	33 (19.1)	26 (15.0)	0.3165	0.1583
Aspirin use*	78 (45.1)	86 (49.7)	0.3890	-0.1023
Non-statin antilipemic*	17 (9.8)	15 (8.7)	0.7105	0.0760
Conc. medications, n (%)				
Antibiotics+	127 (73.4)	123 (71.1)	0.6310	0.0636
GAS drugs+	144 (83.2)	121 (69.9)	**0.0033**	0.4179
Narcotics+	104 (60.1)	88 (50.9)	0.0833	0.2071
Anti-diarrheals+	18 (10.4)	10 (5.8)	0.1125	0.3518
Bowel prep+	48 (27.7)	32 (18.5)	**0.0408**	0.2900
CDI-related medications, n (%)				
Metronidazole+	154 (89.0)	150 (86.7)	0.5100	0.1198
Oral vancomycin+	84 (48.6)	60 (34.7)	**0.0087**	0.3171
Fidaxomicin	3 (1.7)	2 (1.2)	0.6513	0.2268
Probiotics+	52 (30.1)	48 (27.7)	0.6352	0.0621
Healthcare utilization, n (%)				
Prior outpatient visit*	170 (98.3)	168 (97.1)	0.4720	0.2882
Prior inpatient visit*	135 (78.0)	132 (76.3)	0.7008	0.0543
Chronic dialysis therapy*	8 (4.6)	9 (15.2)	0.8035	-0.0683
Residence in LTCF*	22 (12.7)	22 (12.7)	1.0000	0.0000

SMD = Standardized Mean Difference

*Denotes variables included in propensity score

+Denotes variables included in multivariable model following propensity score matching

As a secondary analysis, we evaluated the association between statin use and CDI outcomes in an unmatched cohort that included all CDI patients who met cohort inclusion criteria in a series of logistic regression models that included each outcome as the dependent variable, and 51 covariates (combined covariates from pre- and post-propensity score matching from primary analysis).

## Results

### Cohort description

The overall cohort meeting study inclusion criteria contained 26,149 VHA enrollees diagnosed and treated for CDI, of which 699 (2.7%) were defined as statin users. Overall, the population was predominantly elderly, non-Hispanic, white males (Table 1). Patients were primarily categorized with HCFO-CDI, and the most common concomitant infections were pneumonia and skin infection.

Characteristics of statin users and non-users in the propensity score-matched cohort are provided in Table 1. There were no significant differences in variables entered into the propensity score model. Statin-users were significantly more likely to receive concomitant GAS medications, bowel prep, and oral vancomycin during their encounter compared to non-users and these were controlled for in the logistic regression model. In both the statin user and non-user groups, the majority of patients had an LDL level in a desirable range. Over 90% of patients had a diagnosis of dyslipidemia and nearly 10% had a prior prescription for a non-statin antilipemic agent.

In the matched cohort, statin use was significantly associated with a reduced risk of 30-day mortality (aOR 0.34, 95% CI 0.16–0.72, p = 0.0046) (Table 2). No significant differences were found for inpatient mortality or 60-day recurrence, though a lower proportion of statin-users experienced 60-day recurrence in the unmatched and matched cohorts. In the sensitivity analysis that did not exclude patients who died within 60 days, statin use was also not significantly associated with 60-day recurrence (OR 0.58; 95% CI 0.26–1.32).

**Table 2:** CDI outcomes among statin users and non-users.

	Statin users	Non-users (reference)	aOR (95% CI)	p-value
**Unmatched cohort (statin users, n = 699; non-users, n = 25,450)**				
Inpatient mortality	68 (9.7)	2,376 (9.3)	1.00 (0.55–1.83)	0.9979
30-day mortality	147 (21.0)	5,707 (22.4)	***0*.*55 (0*.*33–0*.*92)***	***0*.*0219***
60-day recurrence*	45 (8.6)	2,565 (13.7)	0.69 (0.38–1.27)	0.2343
**Propensity score-matched cohort (statin users, n = 173; non-users, n = 173)**				
Inpatient mortality	15 (8.7)	12 (6.9)	0.95 (0.32–2.78)	0.9186
30-day mortality	22 (12.7)	35 (20.2)	***0*.*34 (0*.*16–0*.*72)***	***0*.*0046***
60-day recurrence^+^	13 (9.0)	24 (16.6)	0.68 (0.29–1.59)	0.3761

*Excluded patients with 60-day mortality (n = 525 statin users and n = 18,712 non-users)

^+^Excluded patients with 60-day mortality prior to propensity score matching (n = 145 statin users and n = 145 non-users).

## Discussion

In this national cohort of veterans, we found that statin use prior to and during a CDI episode was associated with a lower risk for 30-day mortality compared to non-use. Statin use was not associated with a significant reduction in inpatient mortality or 60-day CDI recurrence. Our study is strengthened by the use of a nationally-representative population and strict control for statin exposures and covariates. The VHA is an integrated healthcare system with pharmacy data available for all prescriptions filled within the system, which helped to ensure no significant lapse in statin therapy prior to the CDI encounter.

Other studies have evaluated the impact of statin use on CDI outcomes, though with varying results. For example, in a retrospective study done by Saliba et al, there was a significantly reduced mortality rate among 669 statin users (13%) and 1219 statin non-users (21%) (p<0.001). These results were very similar to that found in our study, which saw a 30-day mortality rate occurring in 13% of statin-users and 20% of statin non-users (p = 0.0085). However, they only included patients in the outpatient setting and did not adjust for confounding variables, including comorbidities.[19] Another retrospective study of 199 statin users and 750 statin non-users with CDI found no difference (p = 0.583) in 30-day mortality, which occurred in 5.0% of statin users and 4.1% of statin non-users; however, this study also did not account for confounding factors.[20] Similar to our study, they found a lower rate of 60-day CDI recurrence in patients who were on statins (3%) versus those without statin therapy (7.3%) (p = 0.033); however, their comparison was significantly different likely due to a larger sample size utilized. Lastly, a retrospective study of 178 statin users and 321 statin non-users also saw a benefit in statin use in terms of 30-day mortality, as their multivariate analysis found that statin users were more likely to survive than non-users (OR 1.54), though the results were not significant (95% CI 0.850–2.789, p = 0.392).[20, 21]

The lipid-lowering and pleiotropic effects of statins may play a role in CDI in several ways. One of the primary uses of statins is to treat hyperlipidemia, a condition which not only leads to poor cardiovascular outcomes, but can also cause a persistent, generalized inflammatory state of the body.[9, 10, 22, 23] With regard to CDI patients, those with pre-existing gastrointestinal inflammation, such as that seen in patients with inflammatory bowel disease, are associated with worse clinical outcomes, including CDI recurrence and death.[14–17] The anti-inflammatory effects of statins can also be compared to that of long-term aspirin use, as both may improve these outcomes. For example, in the same study by Saliba et al, long-term aspirin use also reduced 30-day mortality rates (OR 0.62, 95% CI 0.43–0.88).[19] Second, cholesterol contributes to the toxin activity of *C*. *difficile* by facilitating toxin binding to cells and is required for toxin penetration into the cells.[24, 25] Therefore, a reduction in LDL cholesterol can potentially reduce toxin penetration and subsequent CDI. As described earlier, statins have numerous beneficial effects.[11] The mechanism responsible for these effects is likely related to a reduction in the synthesis of cholesterol intermediary products which leads to decreased activation of Rho GTPases, ultimately reducing the response of inflammatory intracellular signaling pathways.[11] These pathways are of particular importance in patients with CDI due to the activation of these pathways by toxins A and B.[12, 13]

This study is limited by the use of extracted medical data, and a retrospective cohort study design. All data collection relied on electronic medical records, and no individual chart reviews were performed. Cohort studies might be subject to misclassification bias and confounding by unmeasured variables. Similarly, comorbidities might not be fully captured using administrative codes and cannot be considered equivalent to medical chart reviews. Although several CDI morbidity and mortality risk factors were included in the logistic regression models, additional factors exist that are not included in the analysis. For example, specific *C*. *difficile* ribotypes are associated with increased virulence. Additionally, we were unable to assess all medications a patient was prescribed. While level of CDI severity did not differ between groups, we could not account for the level of treatment received in terms of promptness or aggressiveness; we acknowledge that this may have an effect on survival, and ultimately, these results. Patients could have been on additional agents not accounted for that could modify a patient’s risk for development of CDI as well as a patient’s CDI outcomes. For the recurrence outcome, we analyzed the data both including and excluding patients who died within 60 days and adjusted for potential confounding; however, this may not fully limit the possibility of survival bias in our cohort. Furthermore, the predominantly elderly, male, veteran CDI population might not be representative of all CDI populations, limiting the generalizability of our findings. Finally, we utilized a strict definition for statin use, which substantially limited our sample size of statin-users and resulted in relatively wide confidence intervals for outcomes comparisons.

## Conclusions

In this national study of veterans, CDI patients with statin use prior to and during a CDI episode experienced lower 30-day mortality, but not inpatient mortality, compared to non-users. No significant associations between statin use and CDI recurrence were found. While these data support previous findings reported in the literature, no change in routine care of CDI patients can be recommended at this time. Additional studies on the effects of statins on CDI outcomes may be warranted. Future studies evaluating the impact of statin therapy on other non-cardiac conditions are recommended to further explore the potential benefits of the pleiotropic effects seen with this class of medication.

## Supporting information

S1 Dataset Limited dataset. (XLSX)
